# Editorial: Unsolved problems in congenital diaphragmatic hernia

**DOI:** 10.3389/fped.2023.1177513

**Published:** 2023-03-22

**Authors:** Dick Tibboel, Anne Greenough, Neil Patel, Pietro Bagolan, Thomas Schaible

**Affiliations:** ^1^ Intensive Care, Erasmus MC, University Medical Center Rotterdam, Rotterdam, Netherlands; ^2^ Pediatric Surgery, Erasmus MC, Sophia Children’s Hospital, University Medical Center Rotterdam, Rotterdam, Netherlands; ^3^ Department of Women and Children’s Health, School of Life Course Sciences, King’s College, London, United Kingdom; ^4^ Department of Women and Children's Health, School of Life Course Sciences, King's College London, London, United Kingdom; ^5^ Neonatal Intensive Care Unit, King’s College Hospital, London, United Kingdom; ^6^ Department of Neonatology, Royal Hospital for Children, Glasgow, United Kingdom; ^7^ Neonatal Surgery Unit, Area of Fetal, Neonatal and Cardiological Sciences, Bambino Gesù Children’s Hospital, IRCCS, Rome, Italy; ^8^ Department of Systems Medicine, University of Tor Vergata, Rome, Italy; ^9^ Department of Neonatology, Universitätsklinikum Mannheim, Mannheim, Germany

**Keywords:** newborn, congenital diaphragmatic hernia, ECMO, pulmonary hypertension, follow-up, artificial ventilation, genetics

**Editorial on the Research Topic** Unsolved problems in congenital diaphragmatic hernia

## Introduction

Congenital diaphragmatic hernia (CDH) remains a major congenital anomaly with high mortality and long-term morbidity with many unsolved aspects ranging from the cause to the optimal way of follow-up. Major progress has been made over the last few decades to identify the underlying cause, prenatal therapeutic approaches, neonatal care with regard to optimal ventilatory strategies and treatment of pulmonary hypertension, and evaluation of both surgical and non-surgical morbidities.

Even in the most reputed centers that depend on case mix with only referrals, the availability of extracorporeal membrane oxygenation (ECMO), and a pathophysiological understanding of the role of heart dysfunction, the mortality rate continues to be approximately 20%–30%. Experienced medical teams will acknowledge that in CDH, there are “good and bad years” (like wine) in evaluating mortality in individual clinics.

International collaborations are very important as reflected by the CDH EURO Consortium (guidelines; clinical trials); the International CDH Study Group (epidemiology; risk assessment); DHREAMS (genetics). International therapy guidelines have been published both in Europe and in Canada and Japan. Unfortunately, these provide a low level of evidence even today (1–3).

This important series, Unsolved Problems in CDH, has brought together contributions from established and emerging leaders in the field, from basic science, through fetal therapies, and neonatal pathophysiology to optimized long-term management. Although much remains uncertain and challenges persist, this series provides a “state of the art” of current understanding and identifies key priorities for future research and advancing clinical care in CDH.

## Genetics

Since CDH is a relatively rare disorder with only a few recurrent changes, large cohorts of patients are needed to identify genetic associations. Retrospective whole-genome sequencing of historical patient cohorts will yield valuable data from which the patients of today and tomorrow will profit. Trio whole-genome sequencing has an excellent potential for future re-analysis and data sharing, increasing the opportunity to provide a genetic diagnosis and predict clinical prognosis (Brosens et al.). The success of this effort stresses the importance of collaboration such as within the DHREAMS consortium (http://www.cdhgenetics.com).

Increased insights into the pathogenesis and combination of different congenital anomalies (Gaillard et al.) will be of value to identify specific pathways involved in lung and diaphragm development, together with a profound knowledge of the factors determining (ab)normal lung and diaphragm development (Edel et al.).

Instead of trying to identify “THE CDH gene”, nowadays, disturbances in specific pathways are investigated in more detail (4).

## Prenatal therapy

Advances in prenatal genetic evaluation to guide optimal prenatal counseling have been accompanied by many years of hard work to investigate the impact of fetal intervention. On the other hand, factors associated with the decision for termination of pregnancy (TOP) are additional fetal genetic or anatomical abnormalities and expected severity of pulmonary hypoplasia in left-sided CDH (5).

An international group guided by the Leuven team recently published data on two sets of temporary prenatal occlusion of the human fetal trachea (TOTAL trial) (6, 7). They observed no significant differences in patients with moderate CDH but a significant survival advantage in a group of patients with severe CDH fetuses and who received prenatal tracheal occlusion. This finding will have important consequences for the implementation of prenatal tracheal plugging around the world with debates centered around centralization of this procedure. A high level of and standardization of prenatal diagnosis with either ultrasound or MRI is pivotal for future patient inclusion and for inclusion as a subject of international training courses (8).

## The first breath

Based on animal experiments and evaluation, the first breath and changes in lung inflation and the circulation at transition have resulted in two large randomized trials—Congenital Hernia Intact Cord (CHIC, NCT04429750) (9) and Physiological-based cord clamping for infants with a Congenital Diaphragmatic Hernia (PinC, NCT04373902) (DeKoninck et al.) (10)—to evaluate the effect of delayed, physiology-based cord clamping. Short-term outcomes such as Apgar scores (CHIC) and pulmonary hypertension (PinC) have been selected as primary outcomes. The results are expected in 2–3 years’ time.

International guidelines such as those published over the last decade both by the CDH EURO Consortium and by the Canadian and Japanese study groups advise intubation for every newborn with prenatally diagnosed CDH. Taking into account the potential negative effects of this approach, people have now started questioning this approach, resulting in pilot data on spontaneous breathing in select CDH cases (11). An international collaboration will now evaluate this protocol in more patients on the basis of a proposed algorithm for spontaneous breathing. A value of the observed expected (OE)/lung to head ratio (LHR) of above 50, although arbitrary, is chosen as the cutoff to be included in this study.

## Respiratory support

The optimal method of ventilatory support remains a subject of debate and the same holds true for the application of ECMO in select cases. Although the VICI trial (12) concluded that conventional ventilation (CV) was superior to high frequency oscillation (HFO), this only implies the initial ventilatory mode and also begs the question, “Which CV mode? It is vital that lung-protective ventilation strategies are employed during both initial stabilization and postsurgical repair to avoid ventilator-induced lung damage and oxygen toxicity to prevent further impairment to an already diminished gas-exchanging environment. In this context, clinicians continue to investigate predictive parameters as markers for mortality and morbidity, such as the NeoAPACHE II score and the chest radiographic thoracic area (Amodeo et al.; Weis et al.).

It would be important to evaluate closed-loop automated oxygen control, which is the reinvention of liquid ventilation and heliox therapy in properly designed clinical trials with international collaboration (Williams and Greenough).

Not only differences in mortality but increasingly the incidence and magnitude of chronic respiratory morbidity should be considered primary endpoints in these studies. Objective criteria for lung function both in the acute phase and during child and adulthood are fundamental to any decision to implement future treatment algorithms. In this way, we can establish a state-of-the-art evaluation of the lungs at different time points in life to understand the overall effects on the hypoplastic lungs and the secondary damage by ventilator-induced lung injury (VILI).

## Extracorporeal membrane oxygenation

In select cases, many clinics start ECMO as a rescue therapy, although the overall effects on survival remain debatable. Despite its wide use and decades of experience, the survival rate of CDH patients treated with ECMO, as reported by the extracorporeal life support organization (ELSO), remains unchanged at 50% (13). This is probably due to negative case selection. Individual centers report higher survival rates of up to 70%, such as the Mannheim group (Germany), and can be classified as best practices for this specific treatment modality (14). ELSO data analysis also shows that ECMO improves survival rates in those CDH patients who are most severely affected, but the potential complications of ECMO delivery outweigh the benefits in less severely affected patients. The large variability in ECMO survival rate is determined by preferences such as the mode of ECMO (VV vs. VA), timing of ECMO (early vs. late), patient selection (inclusion criteria variability), surgery (on or after ECMO), supportive cardiac therapy (iNO vs. milrinone), and outcome parameters (alive at decannulation vs. alive at discharge). At present, there is no single test or prognostication that predicts the reversibility of primary pulmonary hypertension of the newborn (PPHN), and the criteria for referral for ECMO are under the process of continued refinement. Therefore, the real contribution of ECMO needs to be investigated in a properly designed RCT using Bayesian statistical approaches.

## The role of the heart in CDH

Postnatal clinical care in CDH has traditionally viewed the lungs as the primary “defective” organs; however, there is growing recognition of the important pathophysiological role played by the heart in CDH.

The coexistence of major cardiac anomalies is an important factor determining survival in CDH and a key consideration in decision making with families of patients in the pre- and postnatal periods.

Pathology specimen and cardiac ultrasound–based studies have additionally identified fetal left ventricular hypoplasia in CDH fetuses. In combination with the established abnormalities of the pulmonary vasculature, this developmental abnormality of the circulation likely contributes to the failed transition at birth, of which variable right and left ventricular dysfunction are key components and determinants of disease severity and outcome (Patel et al.). Although clinical studies have identified the nature of ventricular dysfunction in CDH, the underlying cellular, metabolic, and genetic contributions remain unknown and an important area for investigation.

In the clinical setting, further studies are also required to understand the benefits of routine echocardiographic evaluation of cardiac function, utilizing predetermined and internationally accepted parameters, and the role of this approach in guiding evidence-based therapy of individual patient pathophysiology.

This targeted therapeutic approach, combined with a better understanding of the model-based pharmacokinetic dosing, may lead to a more effective use of well-known and frequently used hemodynamic agents such as iNO, IV sildenafil, milrinone, and PGE1 (Hari Gopal et al.). Unfortunately, the so-called CODINOS trial of iNO and IV sildenafil (15) (Cochius-den Otter et al.) was stopped recently because of a very low inclusion rate and the practical challenges of performing multicenter, investigator-initiated drug studies. The burden of regulatory rules and financial implications underlines the major challenges of performing any clinical trials in CDH.

## Surgery

Closure of the diaphragmatic defect as a semielective surgical procedure is nowadays accepted worldwide. It is an important step in the treatment algorithm incorporating careful planning to prevent intraoperative complications related to hemodynamic instability and recurrent pulmonary hypertension in particular. The defects of most patients with defect sizes of A and B (Boston classification) can be closed primarily without a patch either by an open or a minimally invasive technique, resulting in a change from an abdominal to a thoracic approach. In the latter, CO_2_ inflation is warranted, which might alter the metabolic balance, resulting in pulmonary hypertensive crisis. Less than 10% of newborns will die unoperated almost exclusively of a type D defect (agenesis of the diaphragm) because of a bad prognosis making surgery futile. Importantly, some patients do not classify for surgery already during fetal evaluation. In this group, the main factors are additional fetal genetic or anatomical abnormalities and expected severity of pulmonary hypoplasia in left-sided CDH based on fetal O/E LHR obtained by ultrasound and/or lung volumes by MRI.

As part of a routine longitudinal follow-up, recurrence of the diaphragmatic defect is detected even if cone-shaped patches are used (Zahn et al.; Macchini et al.). The major and most frequently reported downside of minimally invasive surgery in CDH repair is the higher risk of recurrence, reported three- to four-fold higher following an minimal invasive surgery (MIS) approach.

Apart from the original diaphragmatic defect, a long-term follow-up shows the occurrence of hiatal hernia, resulting in gastro-intestinal (GI)- and pulmonary-related morbidity. A longitudinal follow-up with regular radiologic imaging until adolescence is essential to reliably detect recurrence to prevent acute incarceration and chronic gastrointestinal morbidity such as the occurrence of the small bowel obstruction, which may lead to clinical symptoms at any age with serious consequences (Zahn et al.) and impact prognosis.

Optimal timing for repair during ECMO (early vs. late; never on ECMO vs. always; etc.) is still a matter of debate, given the potential bleeding complications during the procedure. No properly designed RCTs are available yet. The same holds true for the choice of the biological or synthetic patch material.

Palliative care for patients, both fetuses and neonates (and their families), who are identified as untreatable because of CDH or associated severe anomalies, is also a future option to consider as part of quality of care.

## Follow-up

The lower mortality rates of CDH patients go hand in hand with the need for paying more attention to the long-term morbidity of different organ systems. Apart from the “classical” organs such as the lungs and the GI tract, a structured long-term follow-up is being offered nowadays by an increasing number of centers. High-volume centers in Rotterdam, the Netherlands, Mannheim, Germany, Rome, and Italy, among others, have published their schedules and results over the years on a variety of aspects (3, 16–18). They use their prospective and highly standardized databases related to the evolution from newborns to adolescence (de Munck et al.; Valfré et al.).

Neurodevelopmental outcome is an important determinant for the future. This has resulted in the concept of “growing into deficit” (19), in which specific higher-executive functions (short-term memory, task performing) have been identified as abnormal and last into adulthood. Prospective longitudinal evaluation combining neuropsychological tests with neuro-imaging are needed for developing a full understanding of these abnormalities and implementation of targeted therapies.

It is very important for comparative analysis that reference values are used on the basis of the respective populations in different countries. Particularly relevant for follow-up are guidelines that are developed in close collaboration between the CDH EURO consortium and ERNICA (European Reference Network for rare Inherited and Congenital Anomalies). ERNICA is a network of expert multidisciplinary healthcare professionals from specialized healthcare providers across Europe. Their aim is to pull together disease-specific expertise, knowledge, and resources otherwise unachievable in a single country.

The involvement of parents in many aspects of care is fundamental to identifying the “real questions” that these families have to contend with on a daily basis. The recent CDH-UK patient journey published as part of this series is a good example of collaboration between healthcare providers and parents to enhance mutual understanding and is of great significance (Power).

## Synthesis

Molecular genetic analysis, combined with an understanding of the developmental pathways of the lungs and diaphragm, will result in an enhanced knowledge of the causes of CDH in individual cases with potential important consequences for fetal interventions. International guidelines for the performance of fetal US/MRI in the context of international collaboration are also pivotal.

Further research is required to identify the optimum method of respiratory support for CDH patients, which is the least damaging to their vulnerable lungs as judged by a reduction in chronic respiratory morbidity. Essential to the success of such research is internationally collaborative randomized controlled trials.

There needs to be an improved understanding of the role of the heart in CDH, combining cellular and clinical studies, incorporating cardiac parameters into fetal predictive scores, and exploring the benefits of pathophysiology-based cardiorespiratory management strategies, from the transitional period onward.

A comparative analysis of internationally available (open access) follow-up databases opens the opportunity for intervention studies aiming to reduce long-term morbidity in different organ systems.

The ultimate aim is to increase survival rates at the lowest level of morbidity. This is a real challenge for all those who are confronted with this very special group of patients who are a significant burden for their parents upon a diagnosis of CDH either during the prenatal stage or as newborns. These patients deserve our lifelong attention and care both from a somatic and from a psychosocial point of view.
